# “Recovery activities are needed every step of the way”—exploring the process of long-term recovery in people previously diagnosed with exhaustion disorder

**DOI:** 10.1186/s40359-024-01756-z

**Published:** 2024-05-06

**Authors:** Ingela Aronsson, Anna Stigsdotter Neely, Carl-Johan Boraxbekk, Therese Eskilsson, Hanna M. Gavelin

**Affiliations:** 1 Department of Psychology, Umeå University, Umeå, 901 87 Sweden; 2 Department of Health, Education and Technology, Luleå University of Technology, Luleå, Sweden; 3 Department of Social and Psychological Studies, Karlstad University, Karlstad, Sweden; 4 Faculty of Medical and Health Sciences, Institute for Clinical Medicine, University of Copenhagen, Copenhagen, Denmark; 5 Department of Diagnostics and Intervention, Diagnostic Radiology, and Umeå Center for Functional Brain Imaging (UFBI), Umeå University, Umeå, Sweden; 6 Institute of Sports Medicine Copenhagen (ISMC) and Department of Neurology, Copenhagen University Hospital Bispebjerg, Copenhagen, Denmark; 7 Department of Public Health and Clinical Medicine, Section for Sustainable Health, Umeå University, Umeå, Sweden; 8 Department of Community Medicine and Rehabilitation, Physiotherapy, Umeå University, Umeå, Sweden

**Keywords:** Exhaustion disorder, Clinical burnout, Recovery activities, Recovery process

## Abstract

**Background:**

Sick-leave rates are high due to stress-related illnesses, but little is still known about the process of recovery from these conditions. The aim of this study was to explore the experiences of the recovery process, 6 to 10 years after treatment in people previously diagnosed with exhaustion disorder (ED), focusing on facilitators and barriers for the process of recovery from ED, and recovery activities experienced as helpful during the recovery process.

**Method:**

Thirty-eight participants (average age: 52 years, 32 females) previously diagnosed with ED were interviewed with semi-structured interviews 6–10 years after undergoing treatment. The interviews were analyzed with thematic analysis.

**Results:**

Three themes resulted from the analysis. The first theme, “A long and rocky road”, summarizes the fluctuating path to feeling better and emphasizes barriers and facilitators that affected the process of recovery, with a focus on external life events and the participants’ own behaviors. Facilitators were changing workplace, receiving support, a reduction in stressors, and changed behaviors. Barriers were a poor work environment, caregiver responsibilities, negative life events and lack of support. The second theme “Recovery activities are needed every step of the way” describes how both the need for recovery activities and the types of activities experienced as helpful changed during the recovery process, from low-effort recovery activities for long periods of time to shorter and more active recovery activities. Recovery activities were described as important for self-care but hard to prioritize in everyday life. The last theme, “Reorienting to a new place”, captures the struggle to cope with the remaining impact of ED, and how internal facilitators in terms of understanding and acceptance were important to reorient and adjust to a new way of functioning.

**Conclusions:**

Recovering from ED is a long and ongoing process where recovery activities are needed every step of the way. Our results highlight the importance of supporting personal recovery and long-term behavioral change, addressing individual stressors that may perpetuate the condition, and adjusting recovery activities according to where the person is in the recovery process.

**Trial registration:**

ClinicalTrials.gov: NCT0073772. Registered on March 8, 2017. This study was pre-registered on Open Science Framework (osf.io).

**Supplementary Information:**

The online version contains supplementary material available at 10.1186/s40359-024-01756-z.

Stress-related illness has gained prominence as one of the leading causes for sick leave in Europe [1, 2]. Stress is a multifaceted concept, encompassing perspectives ranging from biological to social factors [3]. Stress affects health both directly, through mechanisms such as autonomic and neuroendocrine responses, as well as indirectly through changes in health behaviors [3, 4]. Through these mechanisms, stress can give rise to different chronic conditions in addition to specific stress-related diagnoses, such as psychiatric, endocrine and inflammatory diseases [3, 5]. Burnout is one of the most recognized stress-related syndromes, originally associated with work-related stress and not regarded as a clinical condition [6]. More recently, burnout has been suggested to be included in traditional diagnostic classification systems [7] and is at times considered as a mental disorder due to the severity of the symptoms and impact of daily functioning, then referred to as clinical burnout [8]. To date, there is no consensus on how to diagnose clinical burnout and related fatigue-dominated disorders and different overlapping definitions have been used in the literature, including chronic burnout, stress-related exhaustion, job stress-related depression and work-related neurasthenia [9]. In Sweden, exhaustion disorder (ED) has been introduced as a clinical diagnosis equivalent to clinical burnout, characterized by physical and psychological exhaustion and symptoms such as disturbed sleep, cognitive impairment, emotional lability, and somatic symptoms [10], with fatigue being a core symptom reported by patients [11, 12].

The effects of ED can be enduring; ED is usually associated with long sick leave periods of an average of six months [13] and follow-ups ranging from 18 months up to seven years after treatment have shown that a large proportion of former patients experience remaining symptoms [14–16], such as fatigue [16, 17], cognitive difficulties [17], sleep disturbances [18], and lack of recovery after work [16]. The observed long-lasting negative effects of ED on cognition, fatigue, and daily life functioning have led researchers to speculate as to whether these residual symptoms are permanent for some persons [17], or whether the focus of rehabilitation should be adjusted to treating the factors that perpetuate the condition rather than addressing previous stressors [19]. In a scoping review of research on ED, it was concluded that there is still limited knowledge about the course of ED, as well as the optimal mode of rehabilitation [20]. So far, there is limited support for any specific type of treatment, although cognitive behavioral therapy has shown effective for symptom reduction [9, 20].

The process of recovery from mental illness has been suggested to be multidimensional [21] described as a personalized, active and non-linear process, comprising of different stages or phases [22, 23]. Traditionally, research on the recovery process from mental illness has focused on symptom reduction and restoration of functioning, so-called *clinical recovery* [22]. Increasingly, however, the qualitative experience of recovering from mental illness has been highlighted. This can be described as a changed attitude that allows one to live a rich and meaningful life despite one's illness, referred to as *personal recovery* [24]. Thus, personal recovery is not about symptom reduction or reaching an end-goal, rather, it is about finding a helpful approach to live a fulfilling life [23, 24]. To date, few studies have examined the qualitative experience of the process of recovery from ED. In a recent follow-up ten years after treatment, people previously diagnosed with ED described that they were still struggling to understand their illness and to take care of themselves, but that they also had gained an understanding about themselves during the recovery process that helped them change their behaviors and act with more self-care [25]. To be able to be oneself and find time for solitude has also been described as helpful, as well as a supportive environment and being able to balance energy levels during the day [26, 27].

A proposed factor that may maintain clinical burnout is the combination of prolonged stress and disturbed recovery, e.g. restoration of resources [19]. Stress-related ill-health often springs from the lack of recovery in between stressful periods [28, 29] and recovering activities have been shown to be crucial for wellbeing when there is an imbalance between resources and demands [28]. One of the most widely accepted definitions of recovery in this context describes it as a process of psychophysiological unwinding which reduces the strain associated with a stressor [29]. We can further differentiate between recovery activities and experiences; recovery activities refer to the activities during which recovery occurs and recovery experiences to the psychological states associated with recovery, such as psychological detachment or relaxation [30]. Engaging in recovery activities such as physical exercise, socializing, and low-effort activities has been found to have positive effects on well-being, at least in the short-term [30]. Recovery activities have therefore been included as a component in cognitive-behavioral therapy for stress-related disorders [31–33]. However, research on recovery activities in stress-related conditions has mainly been focused on non-clinical populations and little is known about what types of recovery activities are perceived helpful for people previously diagnosed with ED during the recovery process.

In sum, this study focuses on two interrelated concepts that are similar but diverge in their essence: recovery process and recovery activities. Henceforth, the term *recovery process* will be used to refer to the process of recovering from a state of illness (e.g., the process of recovering from ED) and *recovery activities* will be used to refer to activities providing a sense of recovery or restoration of energetic resources.

To date, research on the long-term effects of ED has mainly been conducted using quantitative methods and there is a lack of in-depth understanding of the recovery process from the perspective of persons previously diagnosed with ED. Increased need for recovery activities is a core clinical feature of ED, but the individual's experience of the recovery activities during the recovery process is unknown. The aim of this study was to explore the experiences of the recovery process, 6 to 10 years after treatment in people previously diagnosed with exhaustion disorder (ED), focusing on facilitators and barriers for the process of recovery from ED, and recovery activities experienced as helpful during the recovery process.

## Method

### Design and theoretical underpinning

This qualitative study is based on semi-structured interviews. The interviews were analyzed using a reflexive thematic analysis, chosen due to its flexibility and usefulness to identify broad patterns of meaning across datasets [34, 35]. Grounded in critical realism [36], this study embraced the belief in an independent reality while acknowledging that the interpretation is shaped by individual cultural, social, and political influences. The reporting of the study follows the guidelines from the Consolidated Criteria for Reporting Qualitative Research, COREQ [37].

### Participants and procedure

This study was part of the Rehabilitation for Improved Cognition (RECO) project, a longitudinal randomized controlled clinical trial, which has been described in previous articles [38, 39]. Briefly, the project examined the effects of computerized cognitive training and aerobic training as add-on interventions to a 24-week multimodal stress rehabilitation program (MMR) for people with ED. The added interventions were conducted during the final 12 weeks of MMR. Cognitive function, psychological health and work ability were assessed before, immediately after the interventions and at follow-up one year and 4.5 years after treatment.

All participants had undergone MMR at the Stress Rehabilitation Clinic at the University Hospital in Umeå, Sweden during the years 2010–2013 and were at that time diagnosed with ED according to the Swedish version of the ICD-10 (43.8A), see Additional file 1, Appendix 1. Briefly, the MMR consisted of group cognitive behavioral therapy (CBT), support for balancing physical activity and vocational rehabilitation. Regular visits with a physician were also a part of the MMR. Central elements of the CBT were stress management, recovery activities, strategies for improved sleep and client education with the purpose of increasing awareness of the impact of emotions on stress-related difficulties.

Initially the RECO-project included 132 participants, of which 56 remained at the 4.5-year follow-up (see Gavelin et al. [39] for a complete description of dropouts up until the one-year follow-up and the inclusion- and exclusion criteria in the trial). To recruit participants for the current study, all the previous patients diagnosed with ED (herby referred to as participants) who remained at the 4.5-year follow-up were contacted via letters, followed up by a telephone call by the first author (IA). We aimed to interview all participants who agreed to participate; therefore, no stopping criteria was set. Of the 56 persons contacted, 39 agreed to participate, evenly distributed among previous control- and experimental conditions. Due to ethical reasons, participants were not asked why they declined participation. One participant was excluded due to recent onset of another medical condition. Hence, 38 participants were included in the study. See the flow chart for a visual overview of the process (Additional file 1, Appendix 2). Before the interviews, participants completed self-report forms and a memory aid where they noted key life events that had hindered and helped their process of recovery within the categories work, family, social network, leisure, healthcare, other, things I have done myself (see Additional file 1, Appendix 3). This was done to give the participants an opportunity to recall and reflect upon the years that had passed after they had undergone treatment before coming to the interview, as similar approaches are believed to improve recall [40] and enhance the quality of retrospective memory [41].

To provide a description of the sample, participants filled out self-report forms on psychological health and work ability before the interview. The Shirom–Melamed Burnout Questionnaire (SMBQ) was used to assess level of burnout [42, 43], with a score above 4.4 indicating severe burnout [44]. Moreover, the self-rated Exhaustion Disorder (s-ED) scale was used to assess occurrence of ED-related symptomology [45]. The Hospital Anxiety and Depression Scale (HADS) was used to assess levels of depression and anxiety, with a score above 10 indicating probable occurrence of depression or anxiety [46]. One question from the Work ability index (WAI) were used to assess self-rated work ability (Assume that your work ability at its best has a value of 10 points. How many points would you give your current work ability?), ranging from 0 (cannot work at all) to 10 (work ability at its best) [47], and one question from SF-36 [48] was used to assess self-rated general health (In general, would you say your health is: poor, fair, good, very good, excellent?). One question about perceived recovery from ED was also included (How recovered do you feel after your previous period of exhaustion?) ranging from 0 (not recovered) to 7 (fully recovered).

On average, eight years had passed between the interview and treatment (range 6–10 years). Six participants were men and 32 were women. The age at the time of interview ranged from 34–67 years old (M = 52). Approximately one third of the participants fulfilled the criteria for ED according to s-ED and SMBQ. Approximately one fifth of the participants rated elevated levels of anxiety while only one participant rated elevated levels of depression. Most of the participants rated their health as good (ranging from ‘poor´ to ‘excellent’) and did not feel fully recovered from ED. For details, see Table 1 for participant characteristics.

**Table 1:** Participant characteristics at time of the interview

Variable	Mean (SD) or N (%)
Age^a^	
Mean (SD)	51.9 (7.9)
Range	34–67
Gender^a^	
f/m	32/6 (84%/16%)
SMBQ^b^	
Mean (SD)	3.7 (1.2)
Above cut-off (> 4.4)	10 (27%)
s-ED^b^	
Meet the criteria	13 (35.1%)
HADS-A^c^	
Mean (SD)	7.5 (3.9)
Above cut-off (> 10)	7 (19.4%)
HADS-D^c^	
Mean (SD)	4.0 (3.1)
Above cut-off (> 10)	1 (2.7%)
Perceived work ability^c^	
Mean (SD)	7.1 (1.6)
Perceived general health^b^	
Excellent/Very good	8 (21.6%)
Good	22 (59.5%)
Fair/Poor	7 (18.9%)
Perceived degree of recovery from ED^b^	
1–2 (not recovered)	0 (0%)
3–5	30 (81.1%)
6–7 (fully recovered)	7 (18.9%)
Percentage working hours^d^	
0–24%	0 (0%)
25–49%	1 (2.6%)
50–74%	2 (5.3%)
75–99%	5 (13.2%)
100-%	22 (57.9%)
Retirement	2 (5.3%)
Missing	6 (15.8%)

Number of respondents varies for different variables due to missing data

*SMBQ* Shirom-melamed burnout questionnaire, *s-ED* Self-rated exhaustion disorder, *HADS* Hospital anxiety and depression scale

^a^*n* = 38

^b^*n* = 37

^c^*n* = 36

^d^Based on information noted in the memory aid

### Data collection

All interviews were conducted during the years 2019–2020 by the first author (IA), a female clinical psychologist with ten years of experience working at the Stress Rehabilitation Clinic where the participants had undergone treatment. She had not participated in the treatment of the participants in this study, but she had met some of them during earlier phases in the RECO-project, conducting neuropsychological tests and/or guiding them through cognitive training. Beyond this, the participants had no other knowledge of the interviewer. Most of the interviews were conducted at the Stress Rehabilitation Clinic (N = 31) and some took place over video link (N = 7), due to long travel distance to the clinic. A semi-structured interview template with open ended questions was used (see Additional file 1, Appendix 4). Prompts and follow-up questions were used when needed to help the participant elaborate on the answer, get a better understanding of an answer or clarify a response. The interviews lasted between 52–72 min and were audio recorded and transcribed verbatim by secretaries employed in the project. During the interviews, keywords were noted as field notes. Overall areas addressed in the interviews were the process of recovery from ED with a focus on life events that had facilitated or hindered recovery from ED, recovery activities in everyday life, what activities were experienced as recovering at the time of the interviews and during the recovery process (see the full interview guide in Additional file 1, Appendix 4).

### Analytical approach

The reflexive thematic analysis was carried out through the six steps proposed by Braun and Clarke [35] described below.

During the first phase, familiarization with the transcripts was achieved by reading the interviews while also listening to them (correcting minor mistakes in the transcript). Interviews were then read and re-read, to form initial ideas about essential information in the data. During this re-reading, annotations were made about the essence of each interview. In the second phase, coding was performed inductively and close to the original data, not driven by any theoretical framework. The coding was conducted in the software program NVivo [49]. Early in this phase, two interviews were discussed by members of the research group (IA, HMG and ASN) to share our initial impressions. After the initial coding, the entire dataset was re-examined, comparing the codes with previous annotations about the essence of each interview to assure that all important information was included. In this phase, codes were also reviewed again to ensure consensus was kept over time. In the third phase, preliminary themes were generated in collaboration by members of the research group (IA, HMG and ASN). The development of themes went beyond mere categorization of the data to also interpret the meaning of the information when possible. The preliminary themes were then presented and discussed at a meeting with health care professionals working at the Stress Rehabilitation Clinic as part of a credibility check. At the meeting the head of the department, the section leader and five psychologists participated, all with clinical experience in treatment and relapse prevention of ED. The health care professionals recognized that the study findings aligned well with their clinical observations of patients’ post-treatment. In the fourth phase, the content of each theme was reviewed based on the included coded data extracts and individual themes were reviewed in relation to the entire data set. Some changes were made during this phase, for example merging of two themes. During the fifth phase, the themes were named. Producing the report was not conclusively made in the sixth phase; rather, writing and analyzing were partly conducted simultaneously. Even though we describe the analysis as a linear process, it has been a recursive process moving between the different phases and the wholeness of the report. In the sixth phase, most of the quotes were selected, based primarily on providing an as representative picture as possible of the sample, including as many as possible of the participants' voices. To accentuate readability, we cleaned up quotes by adding punctuations, excluding non-semantic words and for some quotes excluding parts of the quote, marked by […]. Analyses of the data were revised many times in consultation with the second (ASN) and fifth (HMG) author to craft the final analysis.

## Results

**Fig. 1:**
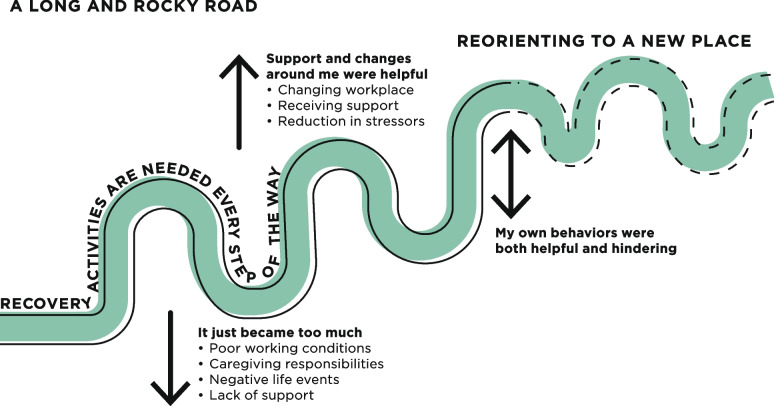
Visual overview of the three themes and their relation. Note. The sub-themes to the theme “A long and rocky road” are included in this figure, as they describe the facilitators and barriers affecting the recovery process over time

The analysis resulted in three themes: “A long and rocky road”, “Recovery activities are needed every step of the way” and “Reorienting to a new place”, each comprising three sub-themes, see Table 2. Together, the themes describe the process of recovery from ED for the participants. The first theme, “A long and rocky road”, summarizes the fluctuating path to feeling better and emphasizes barriers and facilitators that affected the process of recovery, with a focus on external life events and the participants’ own behaviors. The second theme “Recovery activities are needed every step of the way” incorporates recovery activities in the recovery process, describing how both the need for recovery activities and the types of activities experienced as helpful changed during the recovery process, as well as the challenge of incorporating recovery activities in everyday life. The last theme, “Reorienting to a new place”, captures the struggle to cope with the remaining impact of ED, and describes how internal facilitators in terms of understanding and acceptance were important to be able to reorient and adjust to a new way of functioning. Figure 1 shows a model of the three themes and their connectedness, including the sub-themes from the first theme, as these themes and sub-themes all are connected to the process of recovery.

**Table 2:** Overview of themes (bold) and sub-themes

A long and rocky road	Recovery activities are needed every step of the way	Reorienting to a new place
It just became too much	Resting and tranquility was crucial at first	I am better, but not well
Support and changes around me were helpful	Challenging yet attainable to find recovery	It is both sad and strengthening
My own behaviors were both helpful and hindering	Today I don't need to rest in that way to recover	I will never be who I was, and that’s okay

### Theme 1: A long and rocky road

This theme is about the facilitators and barriers that affected the recovery process over time. Participants described the process of recovery from ED as long and ongoing, in many cases several years, where setbacks were common and varied in frequency, duration, and intensity. Over time however, the setbacks became shorter and less severe for most of the participants.

#### It just became too much

This sub-theme describes the experienced external barriers that hindered the recovery process. Poor working conditions and caregiving responsibilities were commonly described, as were negative life events and lack of support.

The participants described poor working conditions with lack of resources such as social support, and high job demands, for instance high responsibility and working in a competitive environment, as hindering for the recovery process, mainly due to lack of time for recovery.

“… (the setback) was of course due to the fact that there was a lot of confusion in the organization and a lot of stress at that time that we might not normally have at my workplace.” – Participant A

Caring for another person, such as a child with special needs or an older parent, also negatively impacted the recovery process for almost half of the participants. This was described as depleting energy in several ways: causing worry and tension, increasing workload at home (e.g., extra meetings with the school or health care), needing to adjust to the needs of the other person and having less room for self-care.

“I’m so exhausted because for my family to work, I have to direct it. I have to arrange, I have to prepare, I have to anticipate the course of events before anything happens and… or well, I don’t have to do it. I do it because it’s worth the effort to do the preparatory work, otherwise it gets much worse afterwards” – Participant B

Negative life events such as conflicts, separations or experiencing a traumatic event were also described as hindering and having a similar impact on the process of recovery as caring for another person: depleting energy, making it harder to focus on self-care and resulting in a higher perceived stress load. Moreover, several participants described that not only the type of stressor but also the total amount of stressors was experienced as hindering.

“I believe that what made me become exhausted again was that a lot happened in the past three years. I went from living alone with my partner to having a child, buying a house that needed renovating, and starting my specialist training. At the same time, we had our second child, so I was never really off-duty […] It just became too much.” – Participant C

A lack of support both at the workplace and outside of work was also described as hindering, resulting in challenges related to a high workload and feelings of loneliness.

#### Support and changes around me were helpful

This sub-theme is about external facilitators the participants had experienced during the recovery process. Commonly mentioned facilitators were changing workplace (either a new profession or the same profession in a different workplace) and receiving support from others. A reduction in stressors in private life was also described as a facilitator.

More than half of the participants had changed workplace after ED. Almost all of them described ED as the main reason for their decision, either due to a poor work environment or because they no longer felt capable to manage their job.

“It was the job that was destructive, that's how it was. I loved it and it was great fun, but there were too many people, too much movement, too many tasks that I felt I wanted to do but didn't have time for […] it was difficult to feel that I wasn't enough. So, it was important, it was great to change job.” – Participant D

Most of the participants who had changed workplace found that their new workplaces were better aligned with their needs after ED, such as having a clearer structure, better routines, a supportive work environment and less stress. This gave them better prerequires for self-care. Some participants considered changing workplace due to suboptimal working conditions but had not yet done so. Adjustments in work assignments were described as helpful by those who stayed at their current workplace as well as by those who had changed workplace. Flexible and adaptable job arrangements, including reduced working hours, facilitated the possibility to engage in recovery activities.

Support from others was described as helpful in different ways during the recovery process, both practical and emotional. Support could come from different sources, such as family, friends, coworkers, management, or the healthcare system. Being understood and treated according to one’s needs was an important aspect of the support, regardless of where it came from.

“They were very nice, both my manager and my co-workers, and that has been an enormous help for me I guess […] they have been quite tough with me but in a good way, saying: 'Now you can't work anymore' (small laugh) 'Sit down' (laugh) 'Go and have a coffee'. A bit like that. They helped me to set boundaries, so that has been an enormous help.” – Participant E

Several of the participants reported helpful changes in their life that had led to a reduction of stressors in their private life, giving room for self-care which positively affected the recovery process. The changes varied in magnitude and character, from small changes such as having more help doing domestic work to larger changes such as moving to a new city, a changed social network or improved health for a person close to them.

“She [a sick relative] has received great help and is back on her feet. So, everything around her has calmed down and, well, is good now. So that's one thing that allows me to relax.” – Participant F

#### My own behaviors were both helpful and hindering

This sub-theme captures how the participants’ own behaviors affected the recovery process, both facilitating and hindering. Many participants described that they struggled to maintain a healthy balance in their lives, often falling back into suboptimal behaviors. They often exceeded their own boundaries, motivated for example by the needs of others, worry, their own desire to perform or difficulties standing up for themselves, reported in quotes like: “I want more than I have the energy to do” (Participant G), “I still overstep my boundaries” (Participant H) and “Sometimes I put myself under quite a lot of pressure” (Participant I). At times, these behaviors caused setbacks. Even though many of the participants still exceeded their limits on occasion, many of them were better at observing their symptoms and adjust their behaviors accordingly.

“I like to do a lot of different things and have a lot of different things going on and sometimes it gets too much. But it will never go as far as it did then.” – Participant A

Some of the participants reported that positive life events in combination with their own behavioral patterns had caused setbacks in their well-being, for example by forgetting to take care of themselves when they were feeling better, or that their enthusiasm made them push themselves too hard. Participants also described challenges to find a balance between doing joyful activities, which in a way gave them energy, while also needing to conserve energy and not activate themselves too much.

Many participants acknowledged their own responsibility to make changes in life and act with more self-care. Improved well-being was associated with changes in behaviors. Setting boundaries and declining activities that exceeded their energy levels were described as helpful strategies.

“Well, I kind of try to keep my pace down and I try to find recovery during the day. That's what I mean, I'm always aware about making sure I'm functioning and that I don't get too stressed” – Participant J

### Theme 2: Recovery activities were needed every step of the way

This theme is about the importance of recovery activities. The need for recovery activities had changed during the recovery process, from being almost the only thing the participants needed to becoming one of many important parts of their daily life. Different types of recovery activities were needed during different times in the recovery process.

#### Resting and tranquility was crucial at first

This sub-theme describes the high need for recovery activities most of the participants had when they felt at their worst, and it is the only theme ranging back prior to the MMR. During what could be considered the acute phase of ED, many described that they spent most of their day recovering in activities such as resting or sleeping and only had energy to be active for short periods of time.

“I needed pure rest […] It did happen at times that I got up in the morning to go to work, had breakfast and then I had to go to bed again because I didn't have the energy for more. So, it was definitely the case that I needed pure rest without any activity at all.” – Participant G

During this phase, the participants expressed a preference for quietness and activities requiring little effort over physical exercise and socializing. To be able to engage in recovery activities such as dining or taking a walk, some needed to rest beforehand. The increased need for recovery activities remained even as the participants began to regain energy and return to their daily activities.

“To be able to work part time, I needed a lot of rest the remaining time. So, when I worked half the day, the other half was a lot of rest. I could rest by leaning against the garage wall or rest in the bed or go out and just sit down by the lake” – Participant K

Participants described that the need for low effort recovery activities could not be disregarded in this stage of the ED process. Had they pushed themselves harder at this time, they would only have felt worse.

#### Challenging yet attainable to find recovery

This sub-theme captures the ongoing challenge to prioritize recovery activities and the conditions that had been helpful to find ways to attain recovery activities and experiences during the recovery process. Several participants, especially those who were the least recovered from ED, described that it was challenging to integrate recovery activities in their daily lives. They reported sporadic engagement in recovery activities by saying they "tried", "should" or "would", whilst these were not a natural part of their everyday life. Some examples were lifted as to why this was difficult: lack of prerequisites, low energy and difficulties understanding their own need for recovery in combination with feeling better.

Having a busy everyday life was mentioned as something that made it harder to focus on recovery activities, and some participants described that they at times forgot to recover when they were feeling better. A few participants described difficulties focusing on recovery activities when their energy was low, especially active recovery activities.

Many of the participants highlighted the importance of having conditions at the workplace that facilitated recovery activities. They found it hard to prioritize recovery activities when the workplace did not have the physical prerequisites for restorative breaks or had a workplace culture that did not encourage breaks during the workday. For example, having a lunchroom that was crowded and noisy or having a high workload was described as poor conditions for recovery.

”We have a staff room, which also serves as a workroom, so you don't sit there on your break and talk about something else. During the break, you go into the classroom and prepare a lesson instead.”—Participant L

Routines were also reported as important to facilitate engaging in recovery activities and some participants expressed that the routines themselves were experienced as recovering. When routines were disrupted, recovery was negatively affected.

#### Today I don't need to rest in that way to recover

This sub-theme describes the changed need for recovery activities during the process of recovery and describes what kind of recovery activities the participants experienced as helpful at the time of the interviews. Almost all participants acknowledged recovery activities as an important ingredient in self-care. At the time of the interviews, a variety of recovery activities were reported. Participants recovered using low effort activities such as silence and solitude, mindfulness, resting/pauses, and spending time in nature. However, less time was needed in low effort recovery activities today compared to when they were feeling the worst. A micro break could be just as effective as a longer break.

“Today I don't need to rest in that way to recover, I can go for a walk and recover […] if I've been active for a long time or had several meetings at work during the day, well, then I get tired, both in my head and in my body. But then if I come home and I eat dinner and so on, then I've got a kind of recovery just through that”—Participant G

Many participants described physical activity, in particular low-intensity physical activities such as walking, as helpful at times during the recovery process, when they no longer were feeling at their worst but still had fluctuating energy levels.

“Slowly but surely, you find something that makes the wheel roll in the right direction again, and to take a walk, to be in nature, it has always been calming and good […] There's not a lot of performance in it, when you walk. Otherwise, I've been running and jogging and so on, but it so easily becomes performance based, so walking is great, it is.” – Participant M

With more energy, physical activity with higher intensity was also experienced as recovering, as were other activities the participants did not have the energy to do when they were feeling their worst, for example creative activities, engaging in activities experienced as interesting or fun, reading or listening to a book, singing in a choir, playing an instrument, traveling, or spending time with others.

“…I had almost no energy or even the desire for it (social contacts) because it just felt hard. And there is a difference now, now I feel that it would be fun (laughter), and then it becomes recovering”- Participant N

However, many of the participants still felt a greater need for recovery activities at the time of the interviews than they had done before ED, needing to balance and adjust the amount of recovery activities depending on their activity load and energy level.

**Fig. 2:**
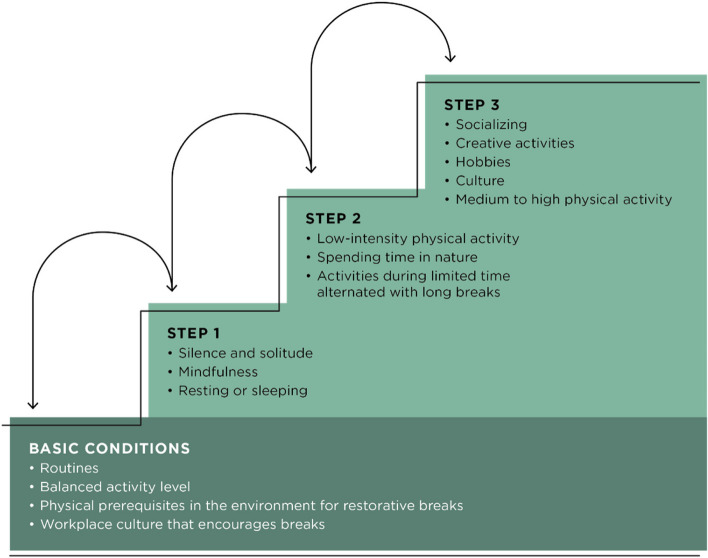
Recovery activity stair. Note. A summary of the descriptions regarding recovery activities during the process of recovery from ED. First, the basic conditions need to be met to create prerequisites to recover. Recovery activities are taken stepwise, where low effort recovery activities such as silence and solitude, mindfulness and/or resting and sleeping are prioritized at Step 1. At Step 2 a balance between low- and high-effort recovery activities are common such as engaging in activities for limited time carefully balanced with low-effort recovery activities or low-intensity physical activity. In Step 3, recovery activities more often include different active recovery activities such as socializing, engaging in hobbies or being physically active at medium to high intensity. Starting point and how much time spent at each step varies as a function of ill-health. Going up or down the steps is related to individual needs.

Taken together, our results regarding recovery activities during the process of recovery from ED are summarized in Fig. 2.

### Theme 3: Reorienting to a new place

This theme captures the struggle to adjust to the remaining impact of ED. It describes what effects ED had on the participants at the time of the interviews, feelings associated with long-term illness and the importance of acceptance and understanding for being able to adjust to a new way of functioning.

#### I am better, but not well

This sub-theme is about the long-term effects of ED. The majority of the participants were feeling better at the time of the interviews compared to directly after the treatment, but almost all still experienced symptoms to various degrees, such as cognitive deficits and stress sensitivity. Many could at times experience a lack of energy and needed to adapt their lives accordingly.

“I have to choose what I do during a day, usually I have to remove something. I have to plan my weeks, if we're going to do something on the weekend, I have to think about how much I'm going to do before that, or if need to rest to be in a good condition. I have to be very careful with what I eat, physical fitness, exercise, stuff like that. How many people I spend time with, I choose not to see people, choose not to have so much mobile and computer time, not to watch too much TV, I read instead. I make sure to go to bed early some evenings. So, all these things that make it work, that's what makes me manage life.” – Participant D

Several participants described that ED had changed them. From being able to push themselves hard and having a high stress threshold, they now had limited strength and endurance. Some described feeling more vulnerable and found it harder to handle obstacles in life. They described that they could not do as much as before and that their energy ran out faster.

"… I will never be-, I have never been as I was before the exhaustion disorder, I will never get there. Superefficient (with a) stress threshold that was, well very high. I do not have that anymore” – Participant O

A few of the participants felt completely recovered after ED reporting “the ED is cured” (Participant P), and “I have never been in a better place than now” (Participant Q), whereas others described a decline in well-being with more frequent and intense symptoms.

#### It’s both sad and strengthening

The central focus of this sub-theme are the feelings associated with long-term illness. Some participants described going through ED as strengthening, as it had helped them learn strategies to cope with other challenging life events and to renavigate and create a life in accordance with their true self.

“But the advantages outweigh the disadvantages. I am a better parent, I am a better leader, and so, I am a better fellow human being than I could have been before.” – Participant R

More common, however, was a remaining feeling of sorrow for still not feeling well and having lost their energy or specific functions such as their good memory. Several participants described that they at times missed their previous way of functioning, even though they could see the benefits of their new approach to life.

“It can be sad as well because, I still identify myself as the one who is always there, everywhere. So, it's like a sorrow in a way, to not be like that anymore, to not have the energy to do it all. At the same time, it's also good to just realize that's how it is, to let it be, to accept it as it is.” – Participant S

Several of the participants did not believe that they would ever have the same abilities and energy as before. Not functioning in the same way as before and not taking care of themselves in the way they "should" according to what they had learned during treatment gave rise to negative feelings such as shame and self-blame.

“It makes me feel a bit ashamed, at the same time that I know that I can't handle it […] first I feel that I haven’t got the energy for this, then I have a guilty conscience because I couldn't be of any help.” – Participant T

#### I will never be who I was, and that’s okay

The essence of this sub-theme is the internal facilitators that has been helpful during the process of recovery. Many participants described that understanding and acceptance of their own behavioral patterns, their limited energy and greater need for recovery activities was important to be able to act in a self-caring way and make choices in life in accordance with their needs. A new attitude towards themselves, such as being kinder and more self-compassionate was also described as helpful.

“Part of recovering from exhaustion disorder and being able to continue in life is accepting that you are different. I am, well, I am not quite as sharp as I was, not quite as quick-thinking […] I don't perform as well intellectually, and under pressure it's more noticeable […] I try not to get sad, it is what it is […] I will never be who I was, that's just how it is. And it's okay because I'm alive and I'm fine” – Participant U

Participants who had understood and accepted their new way of functioning and been able to adapt life accordingly described that life generally worked well, even though they at times experienced symptoms. They could perform for extended periods, especially if they took care of themselves through recovery activities.

"As the years have passed, I have managed a little more every day and […] in these situations when I really need to perform […] I'm at least as brilliant, if I may say so (laughter) as I once was […] but after that performance I need to rest, because my energy runs out faster today”- Participant V.

## Discussion

The aim of this study was to explore the experiences of the recovery process, 6 to 10 years after treatment in people previously diagnosed with exhaustion disorder (ED), focusing on facilitators and barriers for the process of recovery from ED, and recovery activities experienced as helpful during the recovery process. The analysis resulted in three themes: “A long and rocky road”, “Recovery activities are needed every step of the way” and “Reorienting to a new place”. The recovery process from ED was described as long and comprising of reoccurring ups and downs, impacted by both internal and external barriers and facilitators. The need for recovery activities varied during the recovery process, from low-effort recovery activities for long periods of time in the initial stages of ED to shorter and more active recovery activities in the later phase of ED. Almost all participants still experienced some impact of ED in their daily life, where understanding and acceptance helped them manage life despite their symptoms.

The theme “A long and rocky road” show that the struggle to find and keep balance in life continues years after receiving treatment for ED. The participants described recurring ups and downs in their well-being over time, which related to both external and internal barriers and facilitators combined with the ability to manage these, causing an ongoing challenge to maintain balance in life. The results are in line with Ellbin et al. [25], where the recovery process from ED is described as an ongoing, long-lasting process.

Facilitators during the process of recovery from ED were changing workplace, receiving support, a reduction in stressors, changes in the participants’ own behaviors and understanding and acceptance of their condition. Thus, reducing the demands in life, both at work and in private life, seems important for the process of recovery, as not being recovered from ED has been associated with ongoing stressors [50]. The importance of supportive others to feel better after ED have been reported elsewhere [26, 27, 51] and our results add on to this knowledge by showing that social support is important over time as well. Poor working conditions, caregiver responsibilities, negative life events and lack of support were barriers affecting the recovery process negatively. This is in line with previous research showing that both work- and non-work related stressors are common in ED [50, 52]. The participants described that adjustments in work assignments as well as changing workplace were helpful ways to reduce job strain, which aligns well with the results from Beno et al. [53] who found that patients previously diagnosed with ED had changed workplace to a greater extent than the average worker and that the ones making changes were the ones having the most problematic work environment. Importantly, participants described changing workplace as beneficial for the recovery process, whereas ongoing caregiving responsibilities served as a barrier. The latter aligns with previous findings by Eklöf et al. who showed that caring for a child with special needs was associated with long-term ED [50]. Thus, special consideration may be needed for people in treatment where non-controllable stressors are present such as caregiver responsibilities or when work-related changes are necessary but difficult to attain. Our findings are in line with the double burden hypothesis suggesting that the combination of multiple and taxing roles may increase work strain and adverse health outcomes [54, 55]. The elevated prevalence of ED in women has been attributed, in part, to unfavorable working conditions and life situational factors [56]. Despite more equal household patterns over the past decades, women still spend more time and have the primary responsibility for caregiving and household tasks compared to men and report more work–family conflicts than men [57]. Hence, this may perpetuate a vicious cycle where women are living in an environment making them more vulnerable to ED and recovery thereof.

Our results suggest that for people diagnosed with ED the need for and type of recovery activities differs during the recovery process. Prior work has shown that active leisure activities such as socialization and physical activity are helpful recovery activities and are more important for well-being than passive leisure activities such as watching TV for a healthy population [29, 30]. Our results qualify prior findings by showing that during periods where the participants were feeling their worst, active activities were not experienced as restorative whereas passive leisure activities were. However, as the participants health improved, a wide range of more active recovery activities were perceived as helpful, and while low-effort recovery activities were still described as an important ingredient of self-care, less time was needed for those activities to regain energy. Moreover, previous studies have described a greater need for recovery activities during the most acute state for both ED [26] and burnout [8] and engaging in recovery activities have repeatedly been highlighted as a central aspect for regaining health [8, 33]. Our results add to this knowledge by showing that not only the amount of recovery activities but also the type of recovery activities is important. Over time, many of the participants experienced a decreased need for recovery activities, but importantly, some of the participants that were having a relapse at the time of the interviews described an enhanced need for recovery activities again. Thus, the amount of recovery activities and type of recovery activities needed for self-care is not linear but varies according to circumstances.

During the MMR treatment, client education at that time focused on the importance low effort recovery activities for self-care, and we find the participants successive shift to engage in more active recovery activities interesting. A tentative explanation for this is that low effort recovery activities were used to find clinical recovery, e.g., reduce symptoms, whereas engaging in more active recovery activities were used for finding meaningfulness in life and connectedness with others, building on personal recovery [22]. *Personal medicine* i.e. strategies patients engage in for self-care in addition to what health care has recommended, has been argued to be important to include in rehabilitation [58], which stresses the importance of individually tailored treatments focusing on personal recovery, for example finding meaning in life, connectedness with others and empowerment, in line with other suggestions of a process-based approach to treatment of ED [59].

The participants in our study described challenges to maintain a balanced lifestyle, including prioritizing recovery activities especially when feeling better and the need for recovery activities felt less urgent. This comes as no surprise since prior research has consistently shown that adherence to treatment recommendations such as recovery activities and to implement behavioral change is in general hard [60]. However, the participants also expressed several conditions that facilitated engaging in recovery activities in everyday life such as having routines, a balanced activity level and a helpful environment at work, both in terms of a working atmosphere encouraging and providing a physical environment for restorative breaks. We consider these conditions as an important base for optimizing recovery activities in everyday life. Changes in understanding the need for recovery activities and a changed attitude towards prioritizing themselves appeared to be helpful in this regard. In Fig. 2, we have depicted the participants’ experiences regarding recovery activities as a model of stages of recovery activities in the recovery process in persons with ED. The findings on recovery activities provide insights for therapeutic intervention. First, the results highlight the importance of engaging in recovery activities for self-care throughout the recovery process. Hence, to implement behavioral change and motivational techniques may be helpful to support long-term engagement in recovery activities. Secondly, as the type of recovery activities experienced as helpful varies due perceived health, it may be important to consider where in the recovery process the individual is and tailor activities accordingly to support recovery from ED. Not everyone needs low-effort activities; instead, we encourage exploring what type of recovery activity best suits each person's needs. Given the explorative nature of the current study, this figure is tentative and needs further research.

This study adds to the literature on the long-term effects of ED, showing that many of the participants still experience some impact of ED in their daily life (i.e., not clinically recovered). However, as expected, personal recovery seemed to be more important as to how the participants managed everyday life. Personal recovery and related conceptualizations of the recovery process such as ‘post-burnout growth’ [8] and the description of ‘personal development’ [25], captures aspects of living a better life after ED/burnout due to personal growth and finding one’s own values. The facilitators found in our study (changing workplace, receiving support, a reduction in stressors, changes in the participants’ own behaviors and understanding and acceptance of their condition) overlaps with what has been found to be important factors for personal recovery [22], indicating that the process of recovery could, at least in part, be viewed as a journey towards personal recovery. For example, the concepts *connectedness, meaning in life* and *empowerment* have been described as important for personal recovery [22], where our participants are describing the importance of support from others (in alignment with connectedness), accepting ED (in alignment with meaning in life) and make changes in their life in accordance to their needs, such as changing workplace and reduce stressors (in alignment with empowerment). Hence, the facilitators the participants have experienced during the process of recovery have also been important in finding personal recovery. Clinical and personal recovery are different concepts [24, 61], but they can be interrelated [62]. Moreover, key models on recovery from mental illness commonly describe the process going through various phases [22, 63], and although our analysis did not specifically focus on this aspect, there is nothing that contradicts phases in our findings. The external and internal factors found to influence the recovery process for our participants align well with these models as well as other models of the process of change [64]. What type of help is needed may differ depending on phase [24], also in alignment with the descriptions of our participants.

Enhanced self-understanding, including both insights into the cause of ED and the current way of functioning, as well as a new way to view the world has been described as important factors for the process of recovery from ED in a shorter time frame [26, 51, 65] and we found that this seems to be important in a longer perspective as well. However, our results are showing a more nuanced picture, as participants were describing feelings of sorrow and loss as well as enjoyment of life in relation to enhanced self-understanding. Central to feeling better seem to be the ability to accept changes in one’s function and adjust life accordingly, rather than self-understanding alone. As our results show a reoccurring impact of ED, it is important to note that the path to personal recovery may also encompass difficulties [66]. Thus, even if our participants are considered healthy and/or working full time, a majority are still affected by remaining symptoms in everyday life and therefore need to be extra careful with self-care.

Overall, our findings correspond well with one of the most endorsed frameworks for personal recovery from mental illness [22], which describes recovery from mental illness as an active process that is individual and unique, experienced as a struggle, comprising of stages/phases, non-linear, multidimensional, gradual and for some a life-changing journey. However, this framework has been criticized for having an overemphasis on the ‘internal’, subjective experiences of the recovery process at the expense of the relational aspects [67]. From our results it is evident that the process of recovery from ED is more than a personal journey, as both changes in behaviors and one’s life-situation, the context around the person and the interaction between these two were highlighted as important for the process of recovery. Hence, participants have different premises for making changes in life due to more than personal resources. The importance of context for the process of recovery from ED is evident in other studies as well, for example in that a “single lower income”-group had less improvement during treatment [68] and that persons with caregiving responsibilities were more prone to have long-term exhaustion [50]. Bakker and de Vieres [69] have integrated the concept of self-regulation into the job demands resources theory [70], creating an etiological explanation for burnout proposing that external demands and internal coping strategies interact and create a vicious circle showing how stress can lead to mental illness. We consider if this vicious circle may not only explain the etiology of burnout, but also partly explains how burnout (and/or ED) can be perpetuated. However, Bakker and de Vieres [69] only include work related demands affecting burnout whereas our results show that demands from a person’s private life also are important. Taken together, to understand the process of recovery from ED, we need to incorporate individual, relational and contextual perspectives, as all seems to affect the process.

### Strengths and limitations

There are several strengths of this study; it was conducted in a clinical setting where the participants had confirmed diagnosis of ED at the start of the project, the sample size was relatively large, and a memory aid was used preceding the interviews to support memory of the recovery process. Nevertheless, some limitations should be addressed. Firstly, due to the extensive period studied, it may have been difficult for participants to recall their experiences despite the use of the memory aid, and other life experiences may also have influenced the participants during this period. Moreover, the majority of the participants were women, and although this is representative of the patient group, more research is needed to explore men’s experiences of the recovery process. The aim of the study was broad, covering multiple areas and time spans. While intriguing, it posed a challenge to thoroughly explore all questions in-depth during the interviews. The last seven interviews made in this project were conducted early in the pandemic, and while we constructed a safe environment in accordance with restrictions at time, the influence of this factor on our results remains uncertain. The studied group was heterogenous in terms of feeling recovered from ED as well as in experiences during the recovery process. While this was part of the phenomena under investigation and could be regarded as a strength due to richness in data, it also presents a limitation as we cannot ascertain how this diversity affected the results. All members of the research group have previously worked with this patient group, clinically and/or in research, and thus have an in-depth pre-understanding of the patient group. Nevertheless, this may also enhance the risk of confirmation bias. This has been addressed by thorough discussions in the research group regarding the thematization and guided our choice in analyzing the material close to the original data. Moreover, a credibility check was conducted with clinical professionals in the area. While these procedures strived to reduce the impact of confirmation bias by adding different perspectives on the results, we do acknowledge that the homogeneity of the members of the research group and clinical professionals served as a limitation. Finally, this long-term follow-up study suffered from high attrition-rates, which may lead to a select sample of participants which has to be considered when interpreting the findings and may limit trustworthiness.

## Conclusions and clinical implications

In conclusion, this study shows that recovering from ED is a long and ongoing process where setbacks and remaining symptoms are common. This aligns with previous investigations into patients’ experiences of long-term recovery from ED [25] and we extend those findings by highlighting the importance of viewing facilitators and barriers during the recovery process in the context of individual, relational and contextual perspectives. Our findings further indicate that recovery from ED cannot solely be viewed as a process towards clinical recovery and symptom remission, but rather as an ongoing process that can be understood within the framework of personal recovery; in this context, the ability to accept changes in one’s function and adjust life accordingly were helpful approaches towards well-being.

This study is the first to explore experiences of recovery activities in ED, highlighting the variable need for recovery activities during the process of recovery. Even though recovery activities are needed every step of the way, the type of activity needed changes during the recovery process. Our findings demonstrate the challenges to implementing recovery activities in everyday life, highlighting the importance of supporting long-term behavioral change.

From a clinical perspective, our findings suggest the need to support personal recovery by fostering adequate and flexible coping strategies and by considering where the person is in the recovery process and recommend recovery activities accordingly. The importance of individualizing treatment interventions is evident in our results; different stressors may need to be addressed in different ways. Ever-present stressors such as caregiving responsibilities or non-controllable high work strain may need particular attention.

Future research should further explore our model of stages of recovery activities in the recovery process over time in persons with ED, the recovery activity stair, and evaluate its validity. We also find it important to develop cost effective methods that can be helpful in perpetuate recovery activities over time, as this was challenging for our participants.

### Supplementary Information

Supplementary Material 1: Appendix 1. Diagnostic criteria for exhaustion disorder (F43.8A) according to the Swedish National Board of Health and Welfare (ICD-10-SE) Appendix 2. Flow chart of the process in the RECO-project. Appendix 3. Memory aid filled in by participants before interviews. Appendix 4. Interview guide.

## Data Availability

The datasets generated and/or analyzed during the study are not publicly available due to Swedish law (the Swedish Ethical Review Act: 2003:460) but are available from the authors on reasonable request. For such requests, please contact Anna Stigsdotter Neely or Hanna Malmberg Gavelin.

## Abbreviations

ED: Exhaustion disorder
RECO: Rehabilitation for Improved Cognition (project)
MMR: Multimodal treatment
CBT: Cognitive behavioral therapy
SMBQ: Shirom-Melamed Burnout Questionnaire
s-ED: Self-rated Exhaustion Disorder
HAD: Hospital Anxiety and Depression Scale
ICD: International Statistical Classification of Diseases and Related Health Problems
COREQ: Consolidated Criteria for Reporting Qualitative Research
