# Supplementary material for: “Recovery activities are needed every step of the way”—exploring the process of long-term recovery in people previously diagnosed with exhaustion disorder
Source: BMC Psychol. 2024 May 6;12:248. doi: 10.1186/s40359-024-01756-z (PMC11071262; doi:10.1186/s40359-024-01756-z)
Supplement: Supplementary file 1 — Supplementary Material 1: Appendix 1. Diagnostic criteria for exhaustion disorder (F43.8A) according to the Swedish National Board of Health and Welfare (ICD-10-SE) Appendix 2. Flow chart of the process in the RECO-project. Appendix 3. Memory aid filled in by participants before interviews. Appendix 4. Interview guide. [file 40359_2024_1756_MOESM1_ESM.pdf]

Additional file 1

Appendix 1. Diagnostic criteria for exhaustion disorder (F43.8A) according to the Swedish National Board of Health and Welfare (ICD-10-SE)

A. Physical and mental symptoms of exhaustion during at least two weeks. The symptoms have developed in response to one or more identifiable stressors, which have been present for at least 6 months.

B. Markedly reduced mental energy, manifested by reduced initiative, lack of endurance, or increase of time needed for recovery after mental efforts.

C. At least four of the following symptoms have been present most of the day, nearly every day, during the same 2-week period:

1. Persistent complaints of impaired memory and concentration
2. Markedly reduced capacity to tolerate demands or to perform under time pressure
3. Emotional instability or irritability
4. Insomnia or hypersomnia
5. Persistent complaints of physical fatigue and lack of endurance
6. Physical symptoms such as muscular pain, chest pain, palpitations, gastrointestinal problems, vertigo, or increased sensitivity to sounds

D. The symptoms cause clinically significant distress or impairment in social, occupational, or other important areas of functioning.

E. The symptoms are not due to the direct physiological effects of a substance (e.g., abuse of a drug or medication) or a general medical condition (e.g., hypothyroidism, diabetes, infectious disease).

F. The stress-related disorder does not meet criteria for major depressive disorder, dysthymic disorder, or generalized anxiety disorder.

All criteria with capital letters must be met to set the diagnosis.

If, for or example, criteria for depression are met, exhaustion disorder is to be used as a secondary diagnosis.

Appendix 2. Flow chart

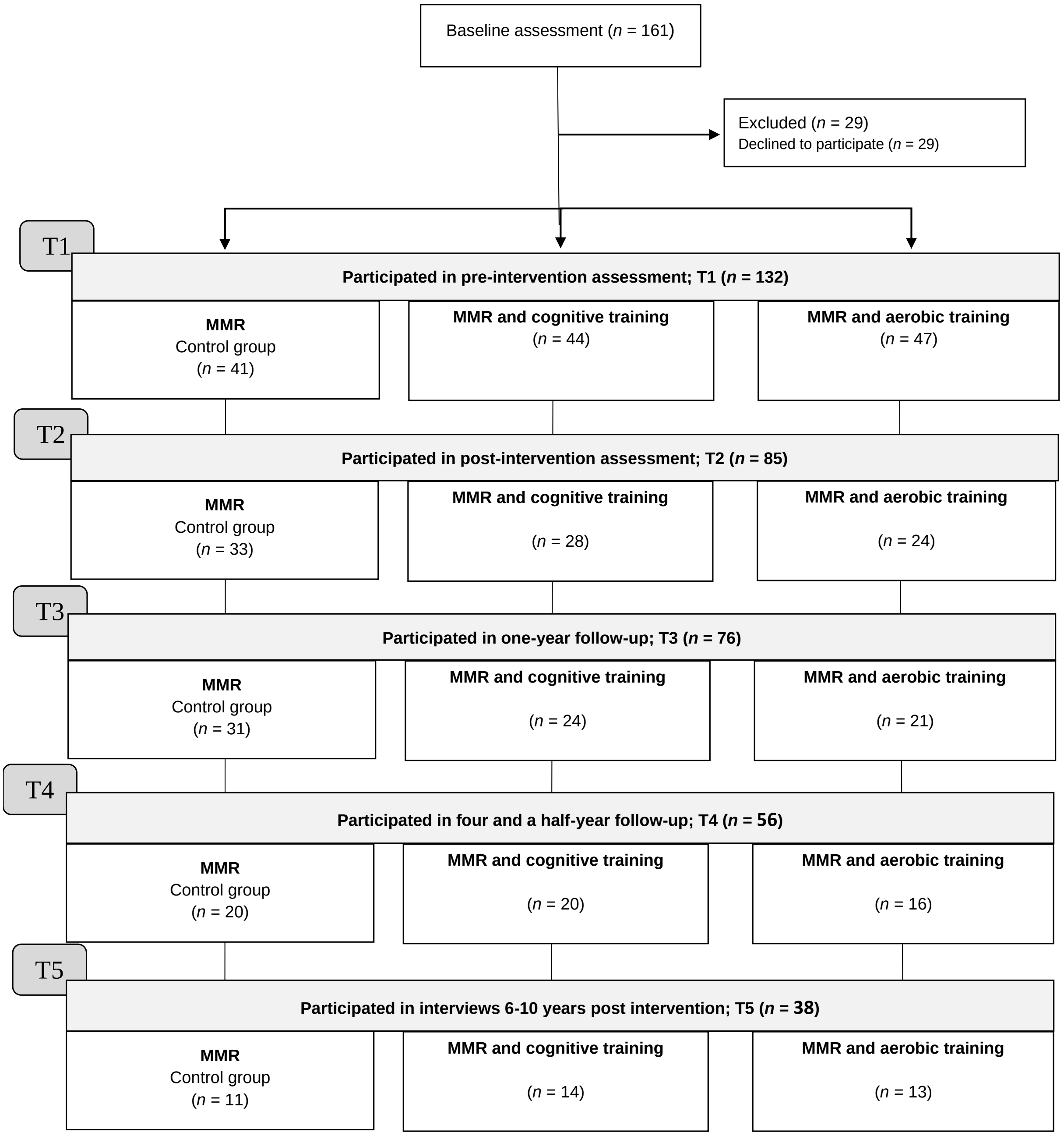

Appendix 3. Memory aid

	2011	2012	2013	2014	2015	2016	2017	2018	2019
**End of treatment**									
% working hours									
**WHAT HAS BEEN HELPFUL FOR YOUR RECOVERY?**									
Work									
Family									
Social network									
Leisure									
Health care									
Other									
Things I have done myself									
**WHAT HAS BEEN HINDERING FOR YOUR RECOVERY?**									
Work									
Family									
Social network									
Leisure									
Health care									
Other									
Things I have done myself									

Appendix 4. Interview guide

Recovery and recuperation

- How are you feeling today in relation to your previous exhaustion disorder?

- Based on the answers to the questionnaire: Could you briefly describe the events that you feel have been helpful for recovery from exhaustion disorder since the end of treatment?
    - If noted, briefly describe what has hindered recovery.

Recovery behaviors and activities

- What are you doing today to take care of yourself in relation to your previous exhaustion disorder?
    - What do you experience as restorative?

- What did you need to do to take care of yourself in relation to your exhaustion disorder when you were the most ill?
    - Has the need for recovery changed?
    - Are the same things restorative today as they were then?
    - Difference in the effect of different recovery behaviors?

- What are your thoughts about the importance of the workplace in creating opportunities for recovery at work?
    - Any adjustments in the workplace that have influenced your opportunities for recovery?
    - Anything missing that could have been helpful (from the employer)?
    - Obstacles/things that have made it difficult?
